# Meat Consumption, Cognitive Function and Disorders: A Systematic Review with Narrative Synthesis and Meta-Analysis

**DOI:** 10.3390/nu12051528

**Published:** 2020-05-24

**Authors:** Huifeng Zhang, Laura Hardie, Areej O. Bawajeeh, Janet Cade

**Affiliations:** 1 Nutritional Epidemiology Group, School of Food Science and Nutrition, University of Leeds, Leeds LS2 9JT, UK; fsaob@leeds.ac.uk (A.O.B.); j.e.cade@leeds.ac.uk (J.C.); 2 Division of Clinical and Population Sciences, Leeds Institute of Cardiovascular and Metabolic Medicine, School of Medicine, University of Leeds, Leeds LS2 9JT, UK; l.j.hardie@leeds.ac.uk; 3 Department of Food and Nutrition, Faculty of Human Sciences and Design, King Abdulaziz University, 21551 Jeddah, Saudi Arabia

**Keywords:** meat consumption, cognitive disorders, dementia, Alzheimer’s disease, cognitive impairment

## Abstract

Cognitive impairment, Alzheimer’s disease, and other forms of dementia are increasing in prevalence worldwide, while global dietary patterns are transitioning to a ‘western type’ with increasing meat consumption. Studies which have explored the associations between cognitive function and meat intakes have produced inconsistent findings. The aim of this systematic review was to explore the evidence linking meat intake with cognitive disorders. Twenty-nine studies were retrieved, including twelve cohort, three case-control, thirteen cross-sectional studies, and one intervention study. The majority (21/29) showed that meat consumption was not significantly associated with cognitive function or disorders. Meta-analysis of five studies showed no significant differences in meat consumption between cases with cognitive disorders and controls (standardized mean difference = −0.32, 95% CI: −1.01, 0.36); however, there was considerable heterogeneity. In contrast, a meta-analysis of five studies showed reduced odds of cognitive disorders by consuming meat weekly or more (OR = 0.73, 95% CI: 0.57, 0.88); however, potential publication bias was noted in relation to this finding. Overall, there was no strong association between meat intake and cognitive disorders. However, the evidence base was limited, requiring more studies of high quality to isolate the specific effect of meat consumption from dietary patterns to confirm these associations.

## 1. Introduction

According to the latest data from the World Health Organization (WHO), around 50 million people worldwide have dementia with an annual incidence of nearly 10 million [1]. Alzheimer’s disease (AD) is the most prevalent type of dementia, clinically characterized by chronic and progressive memory loss, cognitive decline, and other neurodegenerative symptoms [2]. The cause is poorly understood but diet may be a potentially modifiable risk factor [3]. Over recent decades, the consumption of red meat has increased and is forecast to reach 45 Kg per capita per annum as the global annual average consumption by 2030, almost twice as high as during the 1970s [4]. Ecological studies show that in Japan the prevalence of AD rose from 1% in 1985 to 7% in 2008 during the nutrition transition from the traditional Japanese diet to the Western diet [5] characterized by higher red meat intake. National dietary meat supply has also been associated with AD prevalence among ten countries (Brazil, Chile, Cuba, Egypt, India, Mongolia, Nigeria, Republic of Korea, Sri Lanka, and the United States) [6]. However, it is still unknown whether meat intake is linked with cognitive aging or development of AD.

Since meat consumption has been associated with an increased risk of cardiovascular mortality [7], we supposed that it could be a risk factor of cognitive disorders. However, evidence from long-term cohort studies have shown inconsistent associations between consumption of meat and cognitive function. In the Newcastle (UK) 85+ Cohort Study a dietary pattern high in red meat was associated with poor cognition among 791 individuals born in 1921 over a 5-year follow up [8]. Data from 194 cognitively healthy individuals who took part in the Uppsala Seniors cohort study confirmed that a low consumption of meat and meat products was linked with better cognitive scores [9]. However, other studies have shown different results. The Chinese Longitudinal Health Longevity Study (CLHLS) investigated 6911 residents aged 65 or older and found no significant association between higher meat consumption and the risk of dementia after 3 years of follow up [10]. The Maine-Syracuse longitudinal study even showed that higher cognitive scores were prospectively associated with higher intake of meats among 333 participants free of dementia and stroke [11].

As a modifiable factor, diet might potentially support primary prevention related to senile dementia or AD. However, current recommendations for meat intake are unclear in relation to dementia and some countries do not have specific recommended daily allowances. Current information on a healthy diet from WHO only specifies that ‘less than 30% of total energy intake is from fats containing saturated and trans-fats (mainly found in fatty meat and sweet foods)’ [12]. Some key recommendations of the Healthy U.S.-Style Eating Pattern at the 2000-calorie level only stipulate a variety of protein foods including lean meat and limited saturated fats and trans-fats in the 2015–2020 dietary guidelines [13]. Both guidelines refer to limiting saturated fats and trans-fats from meat but do not provide specific daily recommendations for meat intake. In the United Kingdom, a healthy daily portion of red and processed meat was cut down from more than 70 to 90 g (cooked weight) per day in the 2011 report from the Scientific Advisory Committee on Nutrition (SACN) [14], due to ”the link between high consumption and an increased risk of bowel (colorectal) cancer” [15]. In China, 2016 dietary guidelines for the Chinese population recommend ”consumption of an appropriate amount of fish, poultry, eggs, and lean meat, with 280~525 g red meat and poultry per week” [16]. Although these dietary guidelines contain a recommended allowance of meat, few of them have specific recommendations for elderly people, let alone in consideration of cognitive aging. Reasons why these dietary guidelines are limited in specific recommendations on meat may be a lack of reports synthesizing the relevant studies systematically.

The aim of this systematic review was to summarize current evidence with any-type study designs regarding associations between the consumption of meat (exposures) and cognitive function or cognitive disorders (outcomes) among older adults.

## 2. Methods

The review was conducted following the preferred reporting items for systematic reviews and meta-analyses (PRISMA) statement [17] and reported following the meta-analysis of observational studies in epidemiology (MOOSE) [18] checklist (Supplementary Table S1). The study protocol was registered at the International Prospective Register of Systematic Reviews (PROSPERO, registration number: CRD42020173687). 

### 2.1. Search Strategy

A systematic literature search was conducted by investigators using search strategies reviewed by librarians in five databases: EMBASE OVID (1947 onwards), MEDLINE OVID (1946 onwards), Web of Science, Scopus, and the Cochrane Library, up to February 2019. The search was limited to human subjects, using the following terms: ‘meat’ or ‘poultry’ or ‘lamb’ or ‘beef’ or ‘pork’ or ‘mutton’ and ‘cognition’ or ‘dementia’ or ‘Alzheimer’ or ‘AD’ or ‘neurodegeneration’. The specific search terms and strategies are shown in Supplementary Table S2. The search was performed using free text searches in Web of Science and Scopus; and with subject heading searches in EMBASE, MEDLINE and the Cochrane Library databases. An additional search via reference lists of each eligible study was conducted manually to get a more thorough retrieval.

### 2.2. Inclusion and Exclusion Criteria and Screening Process

Articles were included if they fulfilled the following criteria: (1) original research studies; (2) human studies performed in older adults or the elderly (aged 40 years or older) rather than children or youth; (3) studies that provided a description about consumption of meat comprising red meat, processed meat, poultry, but not fish; (4) studies that gave information about methods used for assessing cognitive function, dementia, AD or other cognition-related health outcomes such as cognitive impairment and cognitive decline; (5) studies written in English with full texts available. Studies were excluded according to the following criteria: (1) reviews and book chapters, or secondary-research evidence such as meta-analysis; (2) non-individual studies such as ecological methods; (3) for overlapping studies, the study with the smaller sample size was excluded.

Screening was undertaken by different researchers (H.Z., J.C., and L.H.) who independently assessed texts according to the inclusion and exclusion criteria. Screening results from different researchers were merged and inconsistencies were discussed between the researchers to reach an agreement.

### 2.3. Information Extraction and Quality Assessment

Relevant data and information were extracted into a summary table. Due to the different study designs, an adapted quality assessment scale was created according to the study quality assessment tools (SQAT) for controlled intervention studies, case-control studies, observational cohorts, and cross-sectional studies [19]. Briefly, the adapted scale consists of ten items covering the rationale, sampling, exposure, outcomes, covariates, statistical methods, and potential bias, with detailed instructions for reviewers (Supplementary Table S3). Each item of the scale was given 1 if the answer was “Yes”, or 0 if the answer was “No” or “Not Reported”. Thus, the total quality score ranged from 0 to 10, with higher scores indicating greater quality as scores 0–4 (low quality), 5–7 (moderate quality), and 8–10 (high quality). Data extraction and quality assessment were performed by two reviewers independently (H.Z. and A.O.B.).

### 2.4. Narrative Synthesis and Meta-Analysis

Due to the considerable heterogeneity in study designs, exposure and outcome measures, and analytical methods, it was not statistically appropriate to combine all the included studies in a meta-analysis. Therefore, a formal narrative synthesis on quantitative studies was undertaken according to the reporting guideline of the synthesis without meta-analysis (SWiM) [20]. Briefly, included studies were grouped by study designs and ordered by publication years. Methods of vote counting based on directions of effect and *P* values were applied. Quality assessments on studies included were considered when interpreting findings.

Studies with similar methodologies were pooled in a meta-analysis. For studies reporting the number of participants who consumed any type of meat (fish not included) weekly or more (that is, ‘always’) both in cases diagnosed with cognitive disorders and controls, odds ratios (ORs) and 95% confidence intervals (95% CI) were extracted or calculated to compare the difference in odds of consuming meat weekly or more vs. less frequently between cases and controls. For studies reporting continuous measures such as grams per day or frequency of meat consumed, the standardized mean differences (SMDs) and 95% CI were calculated using Glass’s methods [21] due to very different independent means and SDs of meat intake. This meta-analysis was to compare differences of meat consumption between cases with cognitive disorders and controls. Study heterogeneity was assessed using the Chi^2^ test of homogeneity, where I^2^ statistics of 50% or higher were considered to indicate considerable heterogeneity [22]. Random-effects models (inverse-variance method) were applied to pooling effect sizes. The contour-enhanced funnel plots were created to explore publication bias with Egger’s regression model to detect small-study effects (*P* < 0.05). Meta-analyses and other tests were conducted using Stata version 16.0 (StataCorp).

## 3. Results

### 3.1. Characteristics of Studies and Quality Assessment

**Figure 1:**
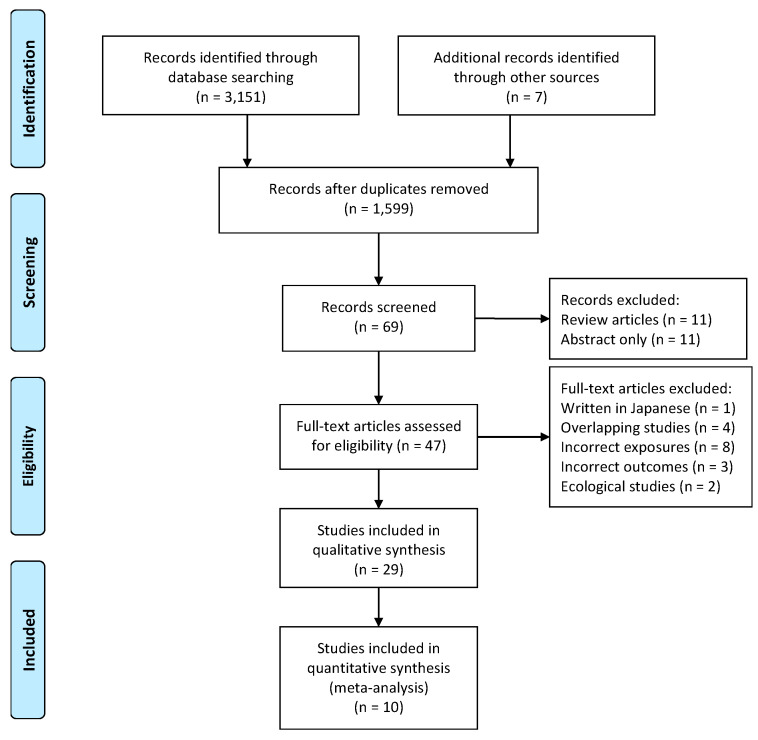
Flowchart of the literature screening by preferred reporting items for systematic reviews and meta-analyses (PRISMA).

In total 3158 records were identified through database and reference list searches. Due to duplication 1559 records were removed, and then 1530 unrelated records were excluded based on titles and abstracts, leaving sixty-nine records. Applying the inclusion and exclusion criteria, a further eleven reviews and eleven records without full texts available were excluded. After reviewing full texts, one record written in Japanese, four records with overlapping studies, two ecological studies, three records with changes of brain structure or β-amyloid (Aβ) deposit as outcomes, and eight records combining meat, fish, and other food together as exposures were excluded, including one paper with an unclear description on meat by Heys et al. (2010) [23] without any reply from two authors contacted. Therefore, twenty-nine eligible records were included in the review: twelve cohort [9,10,11,24,25,26,27,28,29,30,31,32], three case-control [33,34,35], thirteen cross-sectional studies [36,37,38,39,40,41,42,43,44,45,46,47,48], and one intervention study [49] (Figure 1).

Characteristics of included studies are summarized in Table 1. The publication year ranges from 1993 to 2018 and the sample size varies between 48 and 30,484. The mean age of participants was more than 60 years except for two studies, one with a mean age of 52.9 years [46] and one with a range of 40–65 years [43]. Of twenty-nine eligible studies, twenty-four measured consumption of total meat based on a food frequency questionnaire (FFQ) and/or dietary records; one study reported consumption of beef and pork as the exposure [33]; one specified frequencies of use of red meat and sausages as the exposure [31]; two investigated whether participants had habitual intake of red meat with fat or chicken with skin (yes/no) [45,48]; and one intervention study used pork-containing meals as the exposure [49]. Among the studies included, five of them used Alzheimer’s disease and/or dementia as outcomes [24,25,33,39,47], twenty-three measured cognitive function via one or a series of cognitive tests, and one reported both AD and cognitive function [31].

The quality score of each study included is listed in Table 1. Twenty-one out of twenty-nine studies were of moderate quality; four studies were of high quality; and four were of low quality. These latter studies [34,40,47,49] were mainly limited in response or follow-up rate, outcome measures, and adjustment of confounding variables compared with other higher-quality studies, resulting in more caution needed when interpreting evidence. The specific assessment information is shown in Supplementary Table S4.

### 3.2. Observational Evidence

Twenty-eight articles reported observational studies: twelve cohort, three case-control, and thirteen cross-sectional studies. One of the twelve cohort studies reported an inverse association between consumption of meat and meat products and cognitive performance using the seven-minute screening (7MS) test (β coefficient = −0.26, *P* < 0.001) after 5 years of follow-up in Sweden [9]. Three cohort studies observed a protective association. Of these studies, one conducted in France with a 13-year follow-up found that compared with non-consumers, poultry eaters who consumed more than the median (17 g/d) had a reduced risk of recent cognitive decline (adjusted OR = 0.73, 95% CI: 0.58, 0.91), but this was not seen for eaters of offal, red, or processed meat [26]. One cohort study showed that high meat intake was associated with low risk of impairment in memory (adjusted OR = 0.86, 95% CI: 0.75, 0.99) and decision-making (adjusted OR = 0.82, 95% CI: 0.72, 0.93) after a 14-year follow-up in China [32]. The last cohort conducted in the USA also reported high meat intake related to better cognitive scores (β coefficient = 0.062, standard error = 0.012, *P* < 0.001) after an 18-year follow-up [11]. The remaining eight cohort studies did not find any significant associations between meat consumption and cognitive function, or risk of AD and other forms of dementia. These studies had follow-up periods ranging from 4 to 12 years. In addition, all three case-control studies, with one conducted in USA and two in China, did not observe significant associations between meat intake and either Montreal cognitive assessment (MoCA) score or clinical diagnosis of AD.

One of the thirteen cross-sectional studies demonstrated that consumption of meat was negatively related to executive and global cognition function in Poland [44]. One Spanish cross-sectional study used error numbers in tests as outcomes and found that higher meat intake correlated with a greater number of errors incurred (r^2^ = 0.1086, *P* < 0.001); however, the quality of this study was low (score three out of ten) [40]. With dementia or AD as outcomes, one cross-sectional study with higher research quality score showed that meat consumption was associated with increased prevalence of dementia (adjusted PR: 1.19; 95% CI: 1.07, 1.31) in Latin America, China and India [39]; and another Spanish study with a poor research quality found that 47% of AD patients consumed a higher level of meat than the recommended level [47]. The remaining nine cross-sectional studies which were performed in Korea, China, Netherland, Spain, Greece, Australia, the USA, and Brazil did not find any statistically significant associations.

### 3.3. Intervention Study Evidence

The only trial that compared effects of pork-containing meals with chicken-containing meals (control group) was conducted in sixty participants aged 60+ years in Australia [49]. During the 12-week intervention, twenty-nine participants dropped out. The remaining twelve participants in the chicken-eating group had improved verbal learning ability and memory at six weeks (*P* < 0.001), while the nineteen participants in the pork-consuming group did not have significant changes in cognitive function over the 12 weeks; however, the study quality was low (score three out of ten).

### 3.4. Meta-Analysis

**Figure 2:**
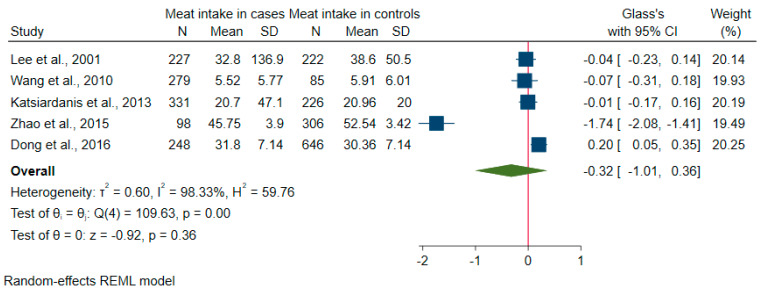
Forest plot of studies with continuous amounts of meat consumed between cases with cognitive impairment and controls for meta-analysis.

There were five studies reporting continuous amounts of meat consumed between cases with cognitive disorders and controls; two reported the eating frequency of meats (e.g., pork, beef and mutton) [41,42], and three reported grams per day of meat or red meat [34,35,36]. In terms of study design and case definition, three were cross-sectional studies with cognitive impairment cases assessed by the mini-mental state examination (MMSE) [36,41,42], two were case-control studies with cases assessed by the MoCA [34,35]. As can be seen in the pooled forest plot (Figure 2), meat consumption in cases with cognitive impairment did not significantly differ from that in controls (overall pooled SMD = −0.32, 95% CI: −1.01, 0.36). However, there is considerable heterogeneity (I^2^ = 98%). The contour-enhanced funnel plot is asymmetric with studies mostly located in the area of *P* > 10% (Supplementary Figure S1), indicating potential publication bias. The Egger’s regression test shows a *P* value of 0.042 indicating that there were small-study effects in the publication bias.

**Figure 3:**
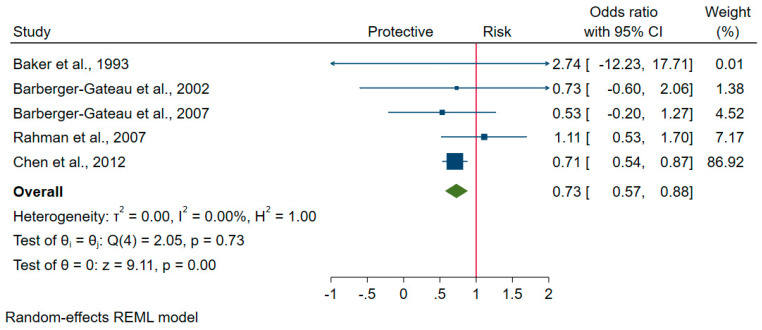
Forest plot of studies reporting odds ratios of those who consumed meat (fish not included) weekly or more (‘always’) vs. less frequently (‘not always’) in cases diagnosed with cognitive disorders compared to controls.

There were five studies with OR values of those who consumed any-type meat (fish not included) weekly or more (that is, ‘always’) vs. less frequently (‘not always’) in cases diagnosed with cognitive disorders compared to controls; four reported consuming meat (e.g., beef/pork/lamb) weekly or more vs. less frequently [24,25,33,38], and one reported ‘always’ vs. ‘not always’ intake of meat [10]. In terms of study design and case definition, four studies comprising two cohort [24,25], one case-control [33], and one cross-sectional [38] study reported AD as cognitive disorders, while one study reporting cases diagnosed with cognitive decline was cohort [10,26,32]. The meta-analysis shows that people with cognitive disorders were 27% less likely than controls to consume meat weekly or more (overall pooled OR = 0.73, 95% CI: 0.57, 0.88) (Figure 3). Heterogeneity is not detected (I^2^ = 0%). Visual inspection of the contour-enhanced funnel plot shown in Supplementary Figure S2 suggests there was a potential publication bias. The quantitative assessment by Egger’s regression test shows no significant presence of small-study effects (*P* = 0.63).

## 4. Discussion

We reviewed current evidence including twenty-eight observational studies and one intervention trial on meat consumption in relation to cognitive function, and cognitive disorders such as cognitive decline and AD. Meta-analysis was only possible on a small number of the studies. The majority of studies included (21/29) showed no statistically significant associations between meat intake and cognitive outcomes: eight out of twelve cohorts, nine out of thirteen cross-sectional, all three case-control studies, and one intervention trial. Interestingly, only one out of the twelve cohorts showed a negative association, while three observed a protective effect of meat intake. In contrast, four out of thirteen cross-sectional studies suggested that high meat intake was associated with poor cognitive performance, and increased risk of AD and dementia. However, cross-sectional studies are limited in terms of potential reverse causality and selection bias.

The meta-analysis of five studies with OR values shows a potentially protective effect of meat intake weekly or more (‘always’) on risk of cognitive disorders; however, there is potential publication bias detected. In addition, a meta-analysis of five studies reporting continuous amounts of meat intake shows no difference in meat consumption between cases with cognitive disorders and controls; however, there is considerable heterogeneity and potential publication bias. It should be noted that in this meta-analysis, one paper by Zhao et al. (2015) reported very narrow standard deviations of meat intake which appears to be an outlier of the funnel plot. Consider that not all studies retrieved from databases were included in these meta-analyses, together with the publication bias and heterogeneity such as in study designs and confounding factors adjusted for, thus more caution should be taken into consideration when evaluating the importance of these findings.

Currently, only a few previous reviews have described associations between meat intake, cognitive function, and dementia. A systematic review (1999) on diet and dementia only reported one study showing a positive but weak association between meat consumption and dementia incidence [50]. Other recent reviews have presented similarly mixed effects of meat consumption on cognitive function, brain structure, and the risk of AD/dementia [51,52]; however, none of them were systematic reviews.

Due to challenges in isolating the effect of meat intake from complex dietary patterns, it is difficult to identify the specific mechanisms underlying the mixed associations between meat consumption, cognitive function and AD/dementia.

Meat, especially lean meat, is high in protein and essential amino acids which are important nutrients for humans. Van de Rest et al. systematically reviewed the role of dietary protein and amino acids in cognitive function among the elderly, and revealed that six out of eight observational studies observed a protective effect of dietary protein and a key role of tryptophan and tyrosine in relation to cognitive function [53]. However, meat contains much saturated fat and cholesterol, which are risk factors for hypertension in relation to an increased risk of AD [54]. In addition, heme accounting for 95% of functional iron in humans is concentrated in red meat; heme deficiency is potentially associated with AD incidence [55], indicating that low intake of red meat may increase the risk of AD.

Secondly, meat consumption may exert its effects on cognitive function or dementia via functional alterations to brain structure assessed by brain imaging biomarkers. Brain volume changes and β-amyloid (Aβ) deposition, as imaging biomarkers, were found to be associated with cognitive impairment and AD measured by magnetic resonance imaging and Positron emission tomography imaging respectively [56,57,58]. Staubo et al. observed an inverse association of red meat intake with cortical thickness among older adults in a cross-sectional study in the United States [59]. Another cross-sectional study showed that lower meat intake was related to larger gray matter volume and total brain volume [60], indicating higher meat intake may be related to reduced brain size and further affect cognitive function in an indirect way. However, Luciano et al. found no associations of meat intake with cortical thickness and volume in the Scottish Lothian birth cohort with following up for 7 years [61]. In addition, the Mayo clinic study of aging (MCSA) observed no associations between meat consumption and Aβ abnormality among 278 older participants [62]. Therefore, whether meat intake is in relation to changes of brain volume or Aβ deposit remains unclear.

Other potential mechanisms may be due to different cooking methods, where some meat cooking methods may produce harmful by-products. For example, cooking methods such as frying and BBQ/grilling may generate benzo[a]pyrene (BaP) in meat, which may induce changes of neurobehavioral function [63,64]. However, these associations are weak and unconvincing at present and more evidence is required.

There are several limitations that need to be taken into consideration when interpreting the findings. Some studies with unpublished non-significant associations, and excluded but relevant studies written in other languages, may result in potential publication bias. Different data types and statistical methods impede the combination of evidence in a meta-analysis. Here, of the twenty-nine included studies, three reported hazard ratios, ten odds ratios, eleven β-coefficients and five used other statistical methods. Another limitation of this review is the heterogeneity of studies included. The differences in cognition measures and diagnostic criteria related to dementia and AD could ultimately affect outcomes. For example, some studies used the Diagnostic and Statistical Manual of Mental Disorders (DSM), third revised version (that is DSM-III-R), to evaluate dementia or AD, while others used the DSM-IV criteria or the National Institute of Neurological and Communicative Disorders and Stroke and the Alzheimer’s disease and Related Disorders Association (NINCDS-ADRDA) criteria, raising issues of heterogeneity. It is important to note various cut-offs, specificity, and sensitivity in different cognitive assessment tools (i.e., MMSE, MoCA). In addition, the use of various cognitive tests to diagnose dementia or AD should be interpreted with caution, since these tests are not gold standard diagnostic criteria. The heterogeneity was not only in definitions of cognitive disorders, but also in measures of meat consumption (e.g., FFQ, 24 h recall, or invalidated dietary questionnaires). Another point should be noted is that less information on covariates is available for most studies included, thus the effect sizes in the meta-analysis are crude, resulting in potential confounding bias. However, we have assessed each study in a comprehensive way and most studies were of moderate or high quality.

Despite the limitations of this review, some strengths should be noted. Firstly, to the best of our knowledge, this study is the first systematic review to specifically explore associations of meat intake with cognitive function, AD, and other forms of dementia. Most recent reviews and meta-analyses assessed effects of specific dietary patterns, such as the Mediterranean diet or the Dietary Approach to Stop Hypertension (DASH) diet, on cognition or dementia [65,66,67,68]; these patterns represent broad nutritional profiles. In contrast, our study focused on the consumption of meat. In addition, this systematic review covered not only Alzheimer’s disease but cognitive function and other forms of dementia. The development of AD is a long-lasting process with a chronic yet progressive decline of cognitive function. It is not clear at which stage of disease development diet, in particular meat intake, may be involved.

## 5. Conclusions

Overall, twenty-nine papers reported an individual effect of meat intake on cognitive function, AD, and other forms of dementia with inconsistent findings, and most of them could not be combined in a meta-analysis. The majority of studies included showed no strong association between meat intake and cognitive disorders. Meta-analysis of five studies suggested that meat intake was protective against cognitive disorders; however, this was limited with representativeness and potential publication bias.

Based on the present systematic review, it is clear that there are numerous challenges with regard to establishing a conclusive association and providing dietary recommendations on meat to the public. The heterogeneity of study designs, exposure, and outcome measures calls for additional research on the association between meat intake and cognitive disorders. Further studies are necessary to clearly isolate the contributions of meat consumption with well-designed study types. Since randomized control trails would be extremely time-consuming and expensive, we therefore recommend the use of existing large-scale and long-term cohort studies with clearer definitions for exposures and outcomes.

## Figures and Tables

**Table 1:** Characteristics of 29 studies included in the systematic review on associations between meat consumption, cognitive function, and dementia.

Author, Year [Ref]	Country, Study Name	Follow-Up, Year	Sample Size (Female/Male)	Age ^1^ (Mean ± SD/Range)	Exposure Measures	Outcomes (Measure Methods)	Effects	Quality Scores
**Cohort studies**								
Barberger-Gateau et al., 2002 [24]	France, PAQUID	7	1416 (Not Reported)	≥ 68	Frequency of consumption of meat	Dementia (MMSE), AD (DSM-III-R)	No significant association between meat consumption and risk of dementia (P-_trend_ = 0.59, adjusted HR = 0.56, 95% CI 0.26–1.20, for weekly consumers).	6
Barberger-Gateau et al., 2007 [25]	France, The Three-City cohort study (3C)	4	8085 (Not Reported)	≥ 65	FFQ including meat	Dementia (neuropsychological tests and DSM-IV), AD (NINCDS-ADRDA)	No association between risk for all cause dementia and meat consumption (*p* > 0.25) adjusted for age.	7
Vercambre et al., 2009 [26]	France, Etude Epidemiologique de Femmes de la Mutuelle Generale de Education Nationale (E3N)	13	4809 (4809/0)	65·5 ± 1·8	208-item FFQ including red meat, offal, processed meat, poultry	Recent cognitive decline (Deterioration Cognitive Observee questionnaire (observed cognitive deterioration), DECO)	High intake of poultry reduced risk of recent cognitive decline (>median consumption vs. no consumption: aOR = 0.73, 95% CI, 0.58–0.91, P-_trend_ = 0.004); but offal, red or processed meat did not.	7
Chen et al., 2012 [10]	China, The Chinese Longitudinal Health Longevity Study (CLHLS)	3	5691 (4302/1389)	82.94 ± 11.03	Frequency of meat intake (pork, beef, mutton, and poultry)	Cognitive decline (MMSE)	Always meat intake (around daily) could reduce the risk of cognitive decline in bivariate regression model (unadjusted OR = 0.71, 95% CI 0.56–0.89, *P* = 0.0029), but no significant associations emerged for meat intake in adjusted models.	6
Samieri, et al., 2013 [27]	USA, Women’s Health Study	4	6174 (6174/0)	71.9 ± 4.1	131-item FFQ including meat	Global cognitive score (telephone adapted MMSE), verbal memory (the East Boston memory test)	No significant association between red and processed meat consumption and mean score of global cognition (P-_trend_ = 0.16) or verbal memory (P-_trend_ = 0.15).	6
Titova et al., 2013 [9]	Sweden, Prospective Investigation of the Vasculature in Uppsala Seniors (PIVUS)	5	194 (93/101)	70	7-day dietary records including amounts of meat	Cognitive score (seven-minute screening, 7MS)	A low consumption of meat and meat products was linked to a better performance on the 7MS test (β coefficient = −0.26, *P* < 0.001).	5
Wengreen et al., 2013 [28]	USA, The Cache County Memory Study (CCMS)	11	3580 (Not Reported)	≥65	142-item FFQ over past year including meat	Cognitive score (modified MMSE, 3MS)	No significant association between increasing quintiles of red and processed meat and higher 3MS scores (P-linear trend = 0.2796).	5
Ashby-Mitchell et al., 2015 [29]	Australia, AusDiab study	12	577 (284/293)	66.07 ± 4.85	101-item FFQ over past year including meat	Cognitive impairment (MMSE)	No association between odds of cognitive impairment and meat consumption (aOR = 1.005, 95% CI 0.964–1.048).	5
Crichton et al., 2015 [11]	USA, The Maine Syracuse Longitudinal Study (MSLS)	18 ± 5.3	333 (Not Reported)	60.5 ± 12.8	37-item FFQ including meat	Cognitive score (the Wechsler adult intelligence scale, WAIS)	Higher WAIS Scores at baseline were prospectively associated with higher intakes of meats (β coefficient = 0.062, se = 0.012, *P* < 0.001).	8
Trichopoulou et al., 2015 [30]	Greece, the European Prospective Investigation into Cancer and Nutrition (EPIC) -Greece cohort	6.6	401 (257/144)	Mean = 74	FFQ including meat	Improved or unchanged score (cMMSE ≥ 0), mildly lower score (cMMSE −4 to −1), substantially lower score (cMMSE ≤ −5)	No significant odds of having mildly lower score (aOR = 1.14, 95% CI 0.89–1.47) or substantially lower score (aOR = 1.09, 95% CI 0.71–1.69) for an increment of one SD of meat intake.	5
Fischer et al., 2018 [31]	Germany, The German Study on Ageing, Cognition and Dementia in Primary Care Patients (AgeCoDe)	4.5	2622 (1712/910)	81.2 ± 3.4	Single-food-questionnaire on frequency of use of red meat and sausages	AD (DSM-IV and ICD-10), memory decline (CERAD neuropsychological assessment battery)	No significant association was detected between frequency of meat and sausage with incident AD (adjusted HR: 1.09, 95% CI 0.94–1.26, *p* = 0.236) or memory decline (adjusted β = 0.01, 95% CI −0.11 −0.14, *p* = 0.845)	9
Zhu et al. 2018 [32]	China, The Shanghai Women’s Health Study and Shanghai Men’s Health Study (SWHS and SMHS)	14.4	30,484 (18,458/12,026)	70–86	FFQ over past year including meat	Questions on memory, and decision-making ability: no, minor, or serious impairments	High red meat intake (fourth quintile: 44.7–64.3 g/d for women, 52.9–75.8 g/d for men) was associated with a lower likelihood of impairments in memory (aOR = 0.86, 95% CI: 0.75, 0.99), and decision-making (aOR = 0.82, 95% CI: 0.72, 0.93).	6
**Case-control studies**								
Baker et al., 1993 [33]	USA	_	72 (50/22)	75.4	Frequency of beef or pork intake	Clinically diagnosed AD cases (McKnann criteria)	No association between the daily or weekly use of beef or pork with a risk for clinically diagnosed AD (aOR = 4.0, CI = 0.30–∞, *p* = 0.37).	5
Zhao et al., 2015 [34]	China	_	404 (Not Reported)	60–90	FFQ including meat	MCI (Montreal cognitive assessment, MoCA)	No difference (*P* > 0.05) in meat intake (pork, beef and mutton) between MCI cases (45.8 ± 3.9 g/d) and controls (52.5 ± 3.4 g/d).	4
Dong et al., 2016 [35]	China	_	894 (604/290)	62.9 ± 5.25	41-item FFQ including meat and poultry	Cognitive score (Montreal cognitive assessment, MoCA)	No significant association was detected between intake of poultry, red meat with MoCA (*P* > 0.05).	5
**Cross-sectional studies**								
Lee et al., 2001 [36]	Korea	_	449 (239/210)	60–83	24 h dietary recall	Cognitive score (MMSE for Korea)	No significant correlations between MMSE score and meat intake (Correlation coefficients: −0.004 for men 0.096 for women)	6
Requejo et al., 2003 [37]	Spain	_	168 (Not Reported)	65–90	7-day food record	Cognitive decline (MMSE)	No significant difference in meat consumption between MMSE ≥ 28 group and MMSE < 28 group with being stratified by age (*p* > 0.1).	5
Rahman et al., 2007 [38]	USA	_	1056 (708/348)	69 ± 8.9	Frequency of consumption of meat	Cognitive decline (mental status questionnaire, MSQ)	No association between risk of cognitive impairment and intakes of meat (aOR = 0.11, 95% CI: 0.67, 1.84).	9
Albanese et al., 2009 [39]	Latin America, China, and India	_	14,960 (Not Reported)	≥65	Frequency of average weekly meat intake	Dementia (the 10/66 diagnostic algorithm)	A less-consistent, dose-dependent, direct association between meat consumption and prevalence of dementia (adjusted PR: 1.19; 95% CI: 1.07, 1.31).	10
Aránzazu et al., 2010 [40]	Spain	_	178 (Not Reported)	65–97	7 consecutive days food record	Cognitive score (short portable mental state questionnaire, SPMSQ)	The intake of meat correlated with a greater number of errors incurred (Correlation coefficient: r^2^ = 0.1086; *p* < 0.001).	3
Wang et al., 2010 [41]	China, Project of Longevity and Aging in Dujiangyan (PLAD)	_	364 (204/160)	93.02 ± 3.01	Frequency of consumption of meat	MCI (MMSE)	No significant association was detected in both unadjusted and adjusted models (aOR = 1.01, 95% CI 0.92–1.10).	7
Katsiardanis et al., 2013 [42]	Greece	_	557 (320/237)	>65	157-item FFQ	Cognitive impairment (MMSE)	No association between meat and meat products with the presence of cognitive impairment (aOR = 0.96, 95% CI 0.81–1.16 for women; aOR = 1.03, 95% CI 0.84–1.27 for men).	6
Crichton et al., 2013 [43]	Australia	_	1183 (751/432)	40–65	215-item FFQ	Cognitive failures questionnaire (CFQ); Memory Functioning Questionnaire (MFQ)	No associations between CFQ score and MFQ score with consumption of meat (*P* > 0.05).	6
Bajerska, et al., 2014 [44]	Poland	_	87 (Not Reported)	≥60	Frequency and potion size of meat and meat products intake over the last month	Global cognitive (MMSE), executive function (cognitive test battery)	The consumption of red meat and meat products was negatively related to executive function (β = −0.02, 95% CI: −0.03–−0.007, standardized β = −0.33, *p* = 0.01) and global cognition (β = −0.02, 95% CI: −0.04–−0.007, standardized β = −0.25, *P* = 0.01).	6
Franca et al., 2016 [45]	Brazil, The EpiFloripa Elderly survey	_	1197 (778/419)	73.9 ± 19.3	Habitual intake of red meat with fat or chicken with skin (yes/no)	Cognition score (MMSE)	No significant association was detected between intake of red meat with fat or chicken with skin and MMSE scores both in women and men (*P* ≥ 0.057).	7
Brouwer-Brolsma et al., 2018 [46]	Netherlands, Nutrition Questionnaires plus (NQplus) study	_	1607 (770/837)	Mean = 52.9	183-item FFQ over past 4 weeks	Semantic memory and language production (letter fluency test, LFT; processing speed (symbol digit modalities test, SDMT); everyday memory (story recall test, SRT)	The meat intake was negatively related to LFT score (β = −0.006, se = 0.002, *p* = 0.007), SDMT score (β = −0.011, se = 0.005, *p* = 0.02), and SRT score (β = −0.003, se = 0.002, *p* = 0.14) in unadjusted model but not in adjusted models.	6
Rocaspana-García et al., 2018 [47]	Spain	_	111 (70/41)	78.5 ± 6.4	45-item FFQ	AD patients diagnosed in hospital	Almost half of the AD patients (46.8%) ate more meat than recommended.	3
Franca et al., 2018 [48]	Brazil	_	400 (288/112)	≥60	Habitual intake of red meat with fat or chicken with skin (yes/no)	Cognition deficit (MMSE)	No significant association was detected between cognitive deficit and intake of red meat with fat (aOR = 1.053, 95% CI 0.568–1.952) or chicken with skin (aOR = 0.952, 95% CI 0.505–1.793).	6
**Intervention studies**								
Charlton et al., 2016 [49]	Australia	12 weeks	31 (Not Reported)	78.0 ± 6.2	Intervention: Pork meals; Control: chicken meals	Cognitive score (cognitive test battery)	No significant cognition change in the pork intervention group over the 12 weeks, while the chicken group had improved verbal learning and memory at six weeks (*p* < 0.001).	4

^1^ Age is when the outcomes were measured.

## Supplementary Materials

The following are available online at https://www.mdpi.com/2072-6643/12/5/1528/s1, Figure S1: Contour-enhanced funnel plot assessing publication bias reporting meat intake levels in cases with cognitive disorders compared to in controls, Figure S2: Contour-enhanced funnel plot assessing publication bias reporting odds ratios (ORs) of meat consumed weekly or more vs. less frequently in cases with cognitive disorders compared to in controls, Table S1: Reporting checklist of meta-analyses of observational studies in epidemiology (MOOSE), Table S2: Searching terms and strategies, Table S3: Quality assessment scale for observational and intervention studies with detailed guidance, Table S4: Quality assessment results of studies included.

## Abbreviations

SD	standard deviation
PAQUID	Personnes Agées QUID epidemiological study of cognitive and functional ageing
MMSE	Mini-mental state examination
AD	Alzheimer’s disease
DSM-III-R	Diagnostic and Statistical Manual of Mental Disorders, third edition, revised
HR	Hazard ratio
CI	confidence interval
FFQ	food frequency questionnaire
DSM-IV	Diagnostic and Statistical Manual of Mental Disorders, fourth edition
NINCDS-ADRDA	National Institute of Neurological and Communicative Disorders and Stroke/Alzheimer’s disease and related disorders association criteria
aOR	adjusted OR
cMMSE	change of mini-mental state examination
ICD	international classification of disease
CERAD	the Consortiumto Establish a Registry for Alzheimer’s Disease
MCI	mild cognitive impairment
PR	prevalence ratio
