# Supplementary material for: Meat Consumption, Cognitive Function and Disorders: A Systematic Review with Narrative Synthesis and Meta-Analysis
Source: Nutrients. 2020 May 24;12(5):1528. doi: 10.3390/nu12051528 (PMC7285210; doi:10.3390/nu12051528)
Supplement: Supplementary file 1 [file nutrients-12-01528-s001.pdf]

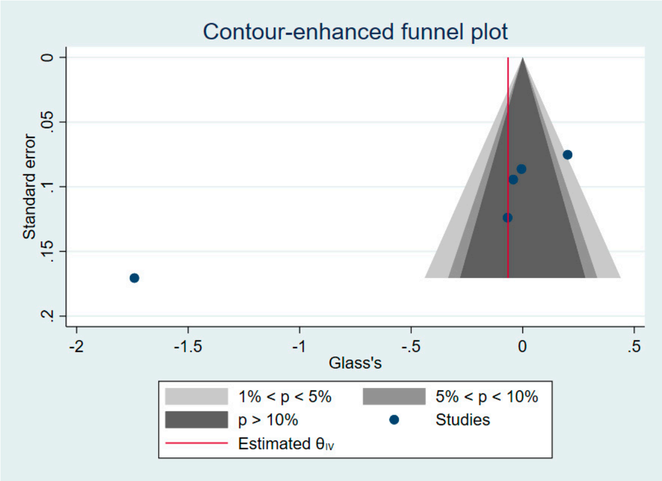

Odds ratios (

Supplementary Figure S1. Contour-enhanced funnel plot assessing publication bias reporting meat intake levels in cases with cognitive disorders compared to in controls.

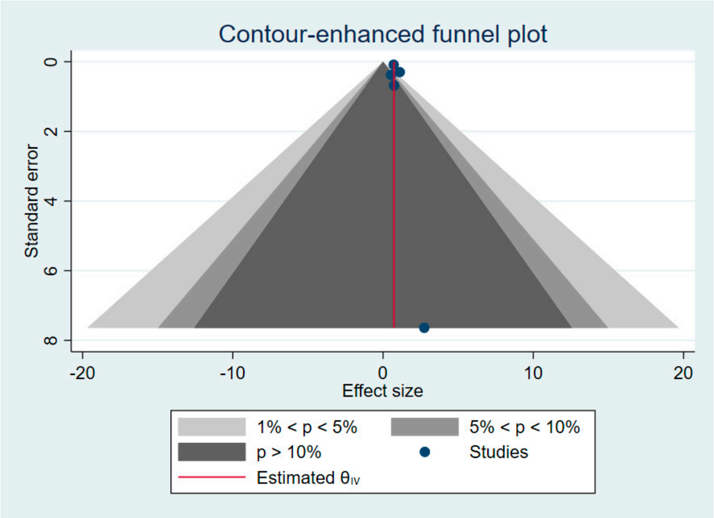

Supplementary Figure S2. Contour-enhanced funnel plot assessing publication bias reporting odds ratios (ORs) of meat consumed weekly or more vs less frequently in cases with cognitive disorders compared to in controls.

Supplementary Table S1. Reporting checklist of meta-analyses of observational studies in epidemiology (MOOSE).

Section/Checklist Item	Reported place
**Reporting of background should include**	
Problem definition	Descripted in the last sentence of the first paragraph of Introduction.
Hypothesis statement	Descripted in the first sentence of the second paragraph of Introduction.
Description of study outcome(s)	Descripted in the last paragraph of Introduction.

Type of exposure or intervention used	Descripted in the last paragraph of Introduction.
Type of study designs used	Descripted in the last paragraph of Introduction.
Study population	Descripted in the last paragraph of Introduction.
**Reporting of search strategy should include**	
Qualifications of searchers (e.g., librarians and investigators)	Descripted in the 2.1 Search strategy.
Search strategy, including time period included in the synthesis and keywords	Descripted in the 2.1 Search strategy.
Effort to include all available studies, including contact with authors	Effort includes free text searches and subject heading searches; reference list searching and contact with authors of paper with unclear description.
Databases and registries searched	Descripted in the 2.1 Search strategy.
Search software used, name and version, including special features used (e.g., explosion)	Did not use search software, but used the EndNote software to manage the records.
Use of hand searching (e.g., reference lists of obtained articles)	Descripted in the 2.1 Search strategy.
List of citations located and those excluded, including justification	The 3.1 section descripted the citations selection process.
Method of addressing articles published in languages other than English	Descripted in the 2.2 inclusion criteria, limited in studies written in English.
Method of handling abstracts and unpublished studies	Descripted in the 2.2 inclusion criteria, limited in studies with full texts available.
Description of any contact with authors	Descripted in the 3.1.
**Reporting of methods should include**	
Description of relevance or appropriateness of studies assembled for assessing the hypothesis to be tested	Descripted in the 2.2 inclusion and exclusion criteria.
Rationale for the selection and coding of data (e.g., sound clinical principles or convenience)	Did not reporting the rationale because the availability of data in the included studies was quite limited, so we tried our best to extract more relevant data.
Documentation of how data were classified and coded (e.g., multiple raters, blinding, and interrater reliability)	Descripted in the 2.4 section.
Assessment of confounding (e.g., comparability of cases and controls in studies where appropriate)	Did not detail it in text, but we assessed the confounding in quality assessment scale in supplementary Table S3 and S4.

Assessment of study quality, including blinding of quality assessors; stratification or regression on possible predictors of study results	Descripted in section 2.3.
Assessment of heterogeneity	Descripted in Figure 2 and 3.
Description of statistical methods (e.g., complete description of fixed or random effects models, justification of whether the chosen models account for predictors of study results, dose-response models, or cumulative meta-analysis) in sufficient detail to be replicated.	Descripted in section 2.4.
Provision of appropriate tables and graphics	Provided in the supplementary tables and other necessary supplementary files.
**Reporting of results should include**	
Graphic summarizing individual study estimates and overall estimate	Figure 2 and Figure 3.
Table giving descriptive information for each study included	Table 1.
Results of sensitivity testing (e.g., subgroup analysis)	Not applicable because few studies were included in the meta-analyses.
Indication of statistical uncertainty of findings	Descripted in the section 3.4.
Reporting of discussion should include	
Quantitative assessment of bias (e.g., publication bias)	Descripted in the first and second paragraphs of the Discussion.
Justification for exclusion (e.g., exclusion of non–English-language citations)	Descripted in the second last paragraph of the Discussion.
Assessment of quality of included studies	Descripted in the second and second last paragraphs of the Discussion.
**Reporting of conclusions should include**	
Consideration of alternative explanations for observed results	Descripted in the first paragraph of the Conclusion.
Generalization of the conclusions (i.e., appropriate for the data presented and within the domain of the literature review)	Descripted in the first paragraph of the Conclusion.
Guidelines for future research	Descripted in the second paragraph of the Conclusion.
Disclosure of funding source	Descripted in the Funding disclosures.

Supplementary Table S2. Searching terms and strategies.

Research components	Searching terms
Meat	Subject heading searching:<br>meat/ meat products/ or processed meat/ or poultry/ red meat/ or beef/ or lamb meat/ or mutton/ or pork/ or rabbit meat/ or veal/ or venison/<br>Keyword searching:<br>meat* or lamb or beef or pork or mutton
Alzheimer's disease	Subject heading searching:<br>Alzheimer disease/<br>dementia/<br>degenerative disease/ (for EMBASE)<br>neurodegenerative diseases/ (for MEDLINE and the Cochrane library)<br>Keyword searching:<br>Alzheimer*<br>dementia<br>neurodegenerati*
Cognition	Subject heading searching:<br>cognition/<br>cognitive disorders/<br>cognitive dysfunction/<br>cognitive defect/<br>cognitive assessment/<br>Keyword searching:<br>cogniti*

* the wildcard character was used to search for all terms that begin with a word; combining search terms were 'OR' between same research components and 'AND' between different research components.

Supplementary Table S3. Quality assessment scale for observational and intervention studies with detailed guidance.

	Criteria	Yes	No	NR*
**1**	Was the research question or objective in this paper clearly stated?			
**2**	Was the sample size clearly defined, calculated and powerful to detect the association of interest?			
**3**	Did this paper describe the eligibility criteria, and the sources and methods of selection of participants?			
**4**	Was the participation rate of eligible persons at least 50% (Response rate or completion rate)? Was loss to follow-up after baseline 20% or less for longitudinal or cohort studies?			
**5**	Were the exposure measures (independent variables) clearly defined, valid, reliable, and implemented consistently across all study participants?			
**6**	Were the outcome measures (dependent variables) clearly defined, valid, reliable, and implemented consistently across all study participants?			
**7**	Were key potential confounding variables measured and adjusted statistically for their impact on the relationship between exposure(s) and outcome(s)? Or matched for case-control studies?			
**8**	Did this paper describe all statistical methods and interpret the results clearly?			
**9**	Did this paper report proportions of missing data and explain how missing data were addressed?			
**10**	Was any potential bias reported and did this paper describe any efforts to address potential sources of bias?			
**Reviewer:**		Total score:		

*NR: not reported

Guidance

Question 1. Research question

Did the authors describe their goal in conducting this research? Is it easy to understand what they were looking to find? This issue is important for any scientific paper of any type. Higher quality scientific research explicitly defines a research question.

Question 2 Sample size

Did the authors present their reasons for selecting or recruiting the number of people included or analyzed? Did the authors describe how to determine the sample size to have enough participants to detect an association if one truly existed?

Questions 3. Study population

Did the authors describe the group of people from which the study participants were recruited, using demographics, location, and time period? If you were to conduct this study again, would you know who to recruit, from where, and from what time period? Is the cohort population free of the outcomes of interest at the time they were recruited? This information is usually found either in descriptions of population recruitment, definitions of variables, or inclusion/exclusion criteria.

An example would be men over 40 years old with type 2 diabetes who began seeking medical care at Phoenix Hospital between 1. 1, 1990 and 12. 31, 1994. In this example, the population is clearly described as: (1) who; (2) where; and (3) when.

Question 4. Participation or follow-up rate

If fewer than 50% of eligible persons participated in the study, then there is concern that the study population does not adequately represent the target population. This increases the risk of bias.

Higher overall follow-up rates are always better than lower follow-up rates, even though higher rates are expected in shorter studies, whereas lower overall follow-up rates are often seen in studies of longer duration. Usually, an acceptable overall follow-up rate is considered 80 percent or more of participants whose exposures were measured at baseline. However, this is just a general guideline. For example, a 6-month cohort study examining the relationship between dietary sodium intake and BP level may have over 90 percent follow-up, but a 20-year cohort study examining effects of sodium intake on stroke may have only a 65 percent follow-up rate.

Question 5. Exposure measures and assessment

Were the exposure measures defined in detail? Were the tools or methods used to measure exposure accurate and reliable? Also, as important is whether the exposures were assessed in the same manner within groups and between groups if applicable.

Here if the meat intake was recorded by 24h dietary recall and FFQs of past 1 month was believed to be relatively accurate, whereas FFQs about the past more than 1 month was not accurate.

Question 6. Outcome measures and assessment

Were the outcome measures defined in detail? Were the tools or methods used to measure outcomes accurate and reliable? Also, as important is whether the outcomes were assessed in the same manner within groups and between groups if applicable.

If the cognitive functions were measured by published known mental scales was believed to be reliable such as: Mini-mental state examination (MMSE), Wechsler Adult Intelligence Scale (WAIS), 10/66 diagnostic algorithm, Montreal Cognitive Assessment (MoCA).

If there are reliable diagnostic criteria for Alzheimer's disease or dementia, such as Diagnostic and Statistical Manual of Mental Disorders 3th/4th edition (DSM-III/IV), National Institute of Neurological and Communicative Disorders and Stroke/Alzheimer's Disease and Related Disorders Association (NINCDS-ADRDA) criteria.

Question 7. Covariates assessment

All key factors that may be associated both with the exposure of interest and the outcome–that are not of interest to the research question–should be controlled for in the analyses. Here the key factors are age, sex, and education level.

Question 8. Statistical analyses

Did this paper describe all statistical methods used clearly, which means you can understand how every single number of interests obtained, including categorized methods on continuous variables, statistical methods to detect the association. Also, the result interpretation was clearly enough to know the exactly association between meat intake and cognitive changes.

Question 9. Missing value

Most studies will have a proportion of missing value, and ignorance under a small proportion or statistical filling using correct methods are reasonable.

Question 10. Potential bias

Did the authors report any potential bias? This information may be reported in the limitation part. Sub-group analysis, sensitivity analysis or other reasonable methods are acceptable.

Supplementary Table S4 Quality assessment results of studies included.

Study	Study design	1	2	3	4	5	6	7	8	9	10	Total
Baker et al., 1993	Case-control study	N	NR	NR	NR	Y	Y	Y	Y	N	Y	5
Lee et al., 2001	Cross-sectional study	Y	NR	Y	NR	N	Y	Y	Y	Y	NR	6
Barberger-Gateau et al., 2002	Longitudinal study	Y	NR	Y	Y	Y	N	Y	Y	N	N	6
Requejo et al., 2003	Cross-sectional study	Y	N	Y	Y	Y	Y	N	N	N	N	5
Barberger-Gateau et al., 2007	Longitudinal study	Y	Y	Y	Y	Y	Y	N	Y	N	N	7
Rahman et al., 2007	Cross-sectional study	Y	Y	Y	Y	Y	Y	Y	Y	N	Y	9
Albanese et al., 2009	Cross-sectional study	Y	Y	Y	Y	Y	Y	Y	Y	Y	Y	10
Vercambre et al., 2009	Longitudinal study	Y	N	Y	Y	N	N	Y	Y	Y	Y	7
Aránzazu et al., 2010	Cross-sectional study	Y	NR	Y	NR	Y	N	N	N	N	N	3
Wang et al., 2010	Cross-sectional study	Y	NR	Y	NR	Y	Y	Y	Y	N	Y	7
Chen et al., 2012	Longitudinal study	Y	N	Y	Y	N	Y	Y	Y	N	N	6
Crichton et al., 2013	Cross-sectional study	Y	N	Y	Y	N	N	Y	Y	NR	Y	6
Katsiardanis et al., 2013	Cross-sectional study	Y	N	Y	Y	N	Y	Y	Y	N	N	6
Samieri, et al., 2013	Longitudinal study	Y	N	Y	Y	N	Y	Y	N	Y	N	6
Titova et al., 2013	Longitudinal study	N	N	Y	Y	Y	N	Y	Y	N	N	5
Wengreen et al., 2013	Longitudinal study	Y	N	Y	Y	N	Y	Y	N	N	N	5
Bajerska, et al., 2014	Cross-sectional study	Y	NR	Y	NR	Y	Y	Y	Y	N	N	6
Ashby-Mitchell et al., 2015	Longitudinal study	Y	N	Y	N	N	Y	Y	Y	N	N	5
Crichton et al., 2015	Longitudinal study	Y	NR	Y	N	Y	Y	Y	Y	Y	Y	8
Trichopoulou et al., 2015	Longitudinal study	Y	N	N	N	N	Y	Y	Y	Y	N	5
Zhao et al., 2015	Case-control study	Y	NR	Y	NR	N	Y	N	Y	N	N	4
Charlton et al., 2016	Intervention study	Y	Y	Y	N	Y	N	N	N	N	N	4
Dong et al., 2016	Case-control study	Y	NR	Y	NR	N	Y	Y	Y	N	N	5
Franca et al., 2016	Cross-sectional study	Y	Y	Y	Y	N	Y	Y	Y	N	N	7
Brouwer-Brolsma et al., 2018	Cross-sectional study	Y	NR	Y	NR	Y	N	Y	Y	Y	N	6
Fischer et al., 2018	Longitudinal study	Y	N	Y	Y	Y	Y	Y	Y	Y	Y	9
Franca et al., 2018	Cross-sectional study	Y	Y	Y	NR	N	Y	Y	Y	N	N	6
Rocaspana-García et al., 2018	Cross-sectional study	Y	N	Y	NR	N	N	N	Y	N	N	3
Zhu et al. 2018	Longitudinal study	Y	NR	NR	Y	N	N	Y	Y	Y	Y	6
